# Interplay of Microbiome, Oxidative Stress and Inflammation in Health and Disease

**DOI:** 10.3390/antiox15020222

**Published:** 2026-02-08

**Authors:** Lourdes Herrera-Quintana, Pablo Iturbe-Sanz, Jorge Olivares-Arancibia, Héctor Vázquez-Lorente, Julio Plaza-Diaz

**Affiliations:** 1 Laboratory of Cellular and Molecular Gerontology, Precision Nutrition and Aging, Madrid Institute for Advanced Studies—IMDEA Nutrition, CEI UAM+CSIC, 28049 Madrid, Spain; lourdes.herrera@nutricion.imdea.org (L.H.-Q.); pablo.iturbe@nutricion.imdea.org (P.I.-S.); 2 Fundación para el Desarrollo y la Innovación Tecnológica (FUNDITEC), C/Faraday 7, Edificio CLAID, Cantoblanco, 28049 Madrid, Spain; 3 AFySE Group, Research in Physical Activity and School Health, School of Physical Education, Faculty of Education, Universidad de Las Américas, Santiago 7500975, Chile; jorge.olivares.a@pucv.cl; 4 Departament de Bioquímica i Biotecnologia, ANUT-DSM (Alimentaciò, Nutrició Desenvolupament i Salut Mental), Universitat Rovira i Virgili, 43201 Reus, Spain; 5 Institut de Recerca Biomèdica Catalunya Sud, 43204 Reus, Spain; 6 Biomedical Research Networking Center for Physiopathology of Obesity and Nutrition (CIBERObn), Institute of Health Carlos III, 28029 Madrid, Spain; 7 School of Health Sciences, Universidad Internacional de La Rioja, 26006 Logroño, Spain

**Keywords:** microbiome, free radicals, redox balance, inflammatory processes, ROS/RNS, redoxomics, microbial metabolites

## Abstract

The human microbiome plays a crucial role in health, being involved in both physiological and pathological processes. The highly dynamic microbiome composition is shaped by different factors, which also may affect host–microbe interactions. Although this relationship is complex and incompletely understood, the interplay between the microbiome, oxidative stress and inflammation is increasingly recognized. Microbial metabolites and specific probiotic strains contribute to maintaining redox homeostasis through multiple pathways, such as regulating the immune system and inflammatory processes or influencing mitochondrial reactive oxygen species production and antioxidant signaling pathways. Oxidative stress and inflammation, in turn, may affect the microbiome by altering microbial diversity and function. These disturbances are believed to create a vicious cycle that further disrupts homeostasis and promotes the appearance of different diseases. This review synthesizes current evidence on the interplay between the microbiome, oxidative stress, and inflammation, highlighting its relevance to both physiological and pathological states.

## 1. Introduction

The human microbiome is a complex ecological community of microorganisms (bacteria, viruses, fungi, eukaryotes and archaea) and their genetic content. Although host–microbe interactions are complex and incompletely understood, the commensal gut microbiome is known to play several beneficial roles in health [1]. Human microbiomes (gut, skin, lung, etc.) have many commons functions, such as immunomodulation, protection against colonization by pathogens, or metabolites production. Additionally, the microbiome may influence redox homeostasis and inflammatory processes through multiple pathways, such as regulating the immune system or influencing mitochondrial ROS production and antioxidant signaling pathways [2]. A disruption in the normal composition, diversity, or function of this microbial community, known as dysbiosis, has been associated with impaired host-health. In this sense, microbiome dysregulation has been reported in several pathologies, and there is broad evidence linking gut-microbiome alterations to intestinal diseases (e.g., Crohn’s disease or colorectal cancer (CRC)) [3].

Moreover, dysbiosis has been associated with oxidative stress, with studies suggesting that the gut microbiome may modulate the concentration of cellular reactive oxygen/nitrogen species (ROS/RNS) via the utilization or production of different metabolites [4]. As consequence of the excessive production of these reactive species, different pathways can be triggered, such as an inflammatory response, immune system activation and DNA damage, also including epigenetic modifications. These processes are believed to create a vicious cycle that further disrupts homeostasis and promotes the appearance of different metabolic and chronic diseases [5].

Microbiome–immune interactions and host inflammatory tone, interconnected by feedback loops mediated through redox balance, should be considered as a dynamic triad of community structure. Hence, a better understanding of microbial metabolism and its relationship with host redox and inflammatory tone has potential clinical and pathological implications [6].

Based on the above, this review synthesizes current evidence on the interplay between the microbiome, oxidative stress, and inflammation, highlighting its relevance to both physiological and pathological states.

## 2. Oxidative Stress and Inflammation

### 2.1. Overview of Oxidative Stress Mechanisms

ROS/RNS are unstable species produced from endogenous (e.g., mitochondria, endoplasmic reticulum, oxidases, etc.) and exogenous (e.g., contaminants, ultraviolet light, ionizing radiation, etc.) sources. These species may contain unpaired electrons, classified as free radicals or non-free radical species. Table 1 summarizes the main species of ROS/RNS [7].

The generation of these species is a natural consequence of evolutionarily conserved metabolic reactions and enzymes activity aimed at energy production [8]. However, ROS/RNS, rather than just metabolites or waste products, play a crucial role in the regulation of numerous physiological processes [7], such as transcription factors (e.g., activator protein 1, p53, nuclear factor kappa B (NF-κB), and nuclear factor erythroid 2–related factor 2 (Nrf2), which is regulated by Kelch-like ECH-associated protein 1 (Keap1)) or signaling pathways (e.g., mitogen-activated protein kinase/extracellular signal–regulated kinase signaling and phosphoinositide 3-kinase–AKT–mechanistic target of rapamycin signaling). Thus, ROS/RNS influence several physiological processes [9]. On the other hand, ROS/RNS are highly reactive molecules, and their concentration may increase very quickly through radical cascade reactions, reaching toxic levels when cells cannot eliminate them efficiently and causing damage to various macromolecules (proteins, nucleic acids and lipids). This condition, with increased and potentially deleterious concentrations of ROS/RNS, is known as oxidative stress [10].

Different mechanisms exist to neutralize the overproduction of ROS/RNS, collectively known as the antioxidant defense system, which tightly controls their concentration and maintains the natural redox balance under physiological conditions. The components of the antioxidant defense system are classified as enzymatic or non-enzymatic. Superoxide dismutase (SOD), glutathione peroxidase (GPx) and catalase (CAT) are the most representative enzymes with antioxidant functions [11,12], while non-enzymatic compounds are low-molecular-weight molecules including vitamin C, vitamin E, flavonoids, carotenoids, and other exogenous molecules. In general terms, antioxidant enzymes are considered the first line of defense against ROS/RNS, with low-molecular-weight antioxidants constituting the second line [13]. Table 2 shows the main components of the antioxidant defense system and their functions.

### 2.2. Overview of Inflammatory Processes

Inflammation is part of the innate and adaptive immune response to injury or infection whose final aim is to restore homeostasis. In normal circumstances, inflammation has two stages: (I) initiation, when the innate immune system is stimulated by pathogens or damage and the inflammatory cascade is activated, and (II) resolution, when the triggering agent disappears and begins an active regulated process (reduction in neutrophil infiltration, clearance of inflammatory cells, tissue repair, etc.) [14]. However, if the stimulus persists, the inflammatory process may become chronic or even dysregulated, leading to fatal consequences such as organ failure. The presence of chronic inflammation is characteristic of several pathologies and low-grade chronic inflammation of aging [15]. 

Damage to epithelial and endothelial cells initiates inflammation by releasing signaling molecules that recruit additional immune cells. The inflammatory response is controlled through tightly coordinated molecular and cellular processes that involve signaling pathways, immune cells, and soluble mediators. At the cellular level, innate immune cells (e.g., neutrophils, macrophages, dendritic cells) are activated and recruited to the inflamed area, releasing cytokines and other mediators that result in amplification of the inflammatory response [16]. Neutrophils are the first cells attracted to a site of injury, acting as central regulators of inflammation, programming antigen-presenting cells to activate T cells and driving the local release of signals that recruit monocytes and dendritic cells. At a molecular level, different stimulus can trigger the inflammatory response through the activation of germline-encoded pattern-recognition receptors, with the toll-like receptors (TLRs) being the most well-studied. As a consequence, intracellular signaling pathways (MAPK, NF-κB, JAK) lead to the production of inflammatory cytokines [17].

Prostaglandins and cytokines are the two main groups of molecules that regulate the inflammatory process. Prostaglandins are produced by cyclooxygenases from polyunsaturated fatty acids and have pro-inflammatory properties. Cytokines comprise several families, including interleukins (about 38 members), and have diverse functions, exerting both pro- and anti-inflammatory effects. Additionally, the inflammatory process is accompanied by a higher production of ROS/RNS since these species act as mediators in the inflammatory response amplification, but also, the inflammatory cascade and the presence of necrotic cells elevate extracellular oxidative stress [15,16].

Once infection or injury is solved, a shift toward an anti-inflammatory state is re-quired to initiate reparative processes. This transition is driven by suppressive signals that reduce pro-inflammatory mediators and leukocyte infiltration and enhance pro-resolution factors such as IL-10 and TGF-β. However, if the insult persists, the inflammation is sustained through several factors (e.g., persistent neutrophil infiltration, deregulated proteolytic activities, upregulation of matrix metalloproteases, etc.) disrupting the healing process and becoming chronic [18].

### 2.3. Consequences of Oxidative Stress and Inflammation

As previously mentioned, the uncontrolled production of ROS/RNS results in oxidative stress, which has detrimental effects on the human body. ROS/RNS react with vital cellular components by several pathways: (I) attacking polyunsaturated fatty acids of cell membranes (lipid peroxidation); (II) modifying amino acid- and fragment peptide chains or causing protein conformational changes (protein oxidation), which results in a loss of structural integrity and function; (III) damaging DNA through strand breaks, base modifications, and cross-linking, causing genomic instability and mutations [19]. Moreover, these cascade reactions may have harmful end products, such as malondialdehyde (MDA) and trans-4-hydroxy-2-nonenal (4-HNE), which are highly reactive end products of lipid peroxidation which may form adducts with DNA and proteins, with elevated levels in different chronic diseases [19,20]. Thus, the maintenance of redox balance is of vital importance for cells.

Furthermore, this redox balance plays a key role in regulating immune function, since immune cells often shift their metabolism from mitochondrial respiration to glycolysis to produce oxidative signals, which are needed for the activation of the pro-inflammatory interleukins and the inflammasome, a multi-protein complex that controls the activation of inflammatory caspases. Moreover, high levels of these reactive species are released to damage and eliminate invading pathogens, serving as a defense tool [21]. Hence, a direct relationship between oxidative stress and inflammation exists, with these processes being potentially deleterious when they are not tightly controlled or properly resolved. In this context, the association of oxidative stress and inflammation with several pathologies has been broadly reported, including neurodegenerative diseases, cancer, cardiovascular diseases or diabetes mellitus, among others [15].

## 3. The Human Microbiome: Composition and Function

**Figure 1:**
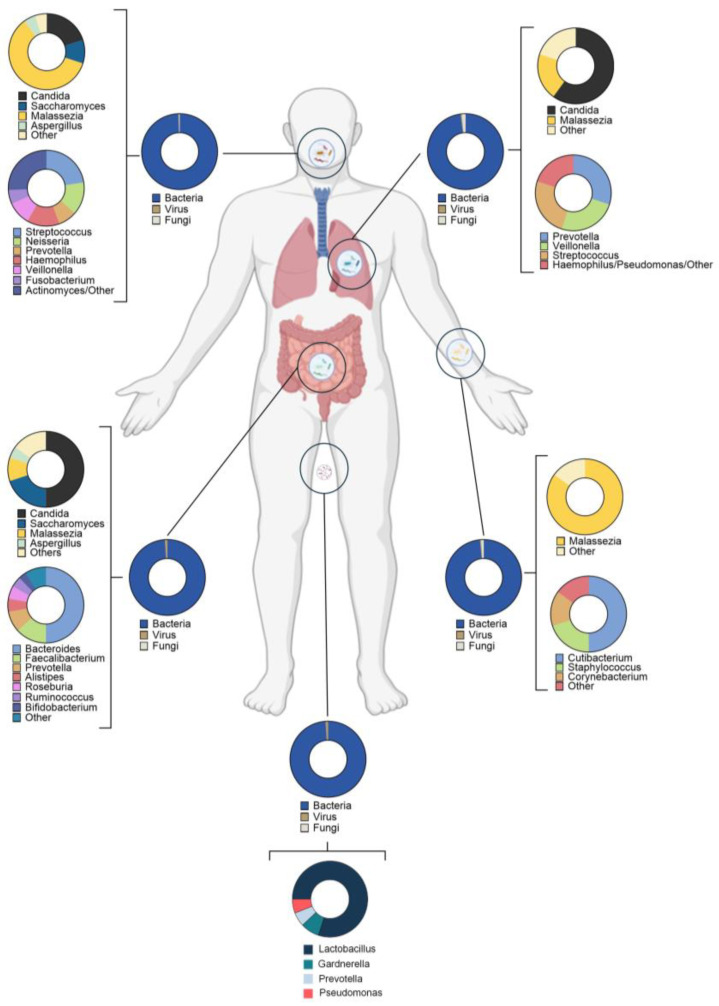
Human microbiome composition and function. Schematic representation of the main human microbiomes (gut, skin, oral, lung, and male and female reproductive tracts) and their predominant microbial taxa [33,52,53,54,55,56,57,58,59].

The human microbiome is a complex ecosystem made up of microorganisms including bacteria, archaea, fungi, and viruses, as well as their metabolic activities resulting from the genes they contain. These communities establish complex relationships with the host, giving rise to homeostasis, which, if disrupted, can have negative consequences for the host. Truly diverse microbiomes can be found depending on the anatomical niche (intestinal, skin, lungs, or oral), each with its own characteristics and predominant populations. The microbiome encodes over 150 times more genes than the human genome, which entails a significant influence on the host metabolism and immunological function [22]. The microbiome composition is highly dynamic, and it is shaped by different factors, including host genetics, diet, lifestyle, age and environmental exposures. The predominant microbial taxa of the gut, skin, oral, lung, and male and female reproductive tracts microbiomes are represented in Figure 1.

### 3.1. The Gut Microbiome

The study of the gut microbiome has retained most of the attention in recent years due to its direct or indirect involvement in host homeostasis, being critical for metabolic and immune regulation and playing a key role in the gut–brain axis [23,24]. The gut microbiome comprises mainly bacteria from the phyla *Bacillota* and *Bacteroidota*, with bacteria from the phyla *Actinobacteriota* and *Pseudomonadota* also abundant. To a lesser extent, archaea, viruses, and fungi can be found in the gut microbiome, thus building a complex ecosystem with interkingdom relationships. Among others, metagenomic and metabolomic analyses have demonstrated the gut microbiome’s influence on host redox balance, especially through metabolites such as butyrate, hydrogen sulfide (H_2_S), and indole derivatives [25]. Additionally, the gut microbiome modulates different signaling pathways, including Nrf2/Keap1, NF-κB, and MAPK through metabolite production. On the other hand, the gut microbiome may influence lipid peroxidation and mitochondrial reactive oxygen species (ROS) production since bacteria contribute to bile-acid biotransformation processes [26]. Lastly, it must be noted that dysbiosis and/or reduced microbiome diversity have been related to different diseases. For instance, oxidative and inflammatory disorders such as Crohn’s disease, obesity, and type 2 diabetes are associated with lower microbiome diversity and increased abundance of *Pseudomonadota* [27,28,29].

### 3.2. The Skin Microbiome

The skin microbiome takes part in the skin’s protective barrier as it colonizes niches susceptible to invasion by pathogenic microorganisms, also playing a key role in lipid metabolism and immune regulation [30,31]. The skin microbiome is entirely dependent on the specific niche in which it is found, since the skin is a complex system that varies in its physical and chemical characteristics [32]. Bacteria and fungi are the predominant kingdoms, with the former being the most abundant. Eukaryotic viruses appear to be more individual-dependent than characteristic of a specific skin-niche [33]. The skin microbiome interacts directly with keratinocytes and immune cells, activating TLRs and nucleotide-binding oligomerization domain-like receptors, then promoting barrier integrity [34]. In sebaceous regions where lipid concentrations are higher, species such as *Cutibacterium acnes* produces porphyrins that can either induce or quench ROS depending on environmental conditions [35]. Furthermore, *Staphylococcus epidermidis* (a commensal member of the skin microbiome) produces lipoteichoic acid which seems to modulate skin inflammation via TLR2/3 [36].

### 3.3. The Lung Microbiome

For many years, healthy lungs have been assumed to be completely sterile [37], but evidence has demonstrated that they house a diverse ecosystem composed of bacteria and, to a lesser extent, fungi and mycobacteria. Bacteria from the genera *Prevotella*, *Veillonella*, and *Streptococcus* have been identified in healthy subjects, where they play a role in maintaining homeostasis and protecting against pathogenic bacteria [38,39]. The lung microbiome plays a fundamental role in redox homeostasis due to the production of short-chain fatty acids (SCFAs), the main metabolites produced during dietary fiber fermentation in the gastrointestinal tract, and in regulating different processes, such as the modulation of alveolar macrophage activity [39]. Moreover, dysbiosis in the lower respiratory tract has been associated with pathologies such as chronic obstructive pulmonary disease and asthma, both characterized by oxidative stress and inflammation [40].

### 3.4. The Oral Microbiome

The oral microbiome is the second most diverse human microbiome, after the intestinal microbiome. The microorganisms that compose it inhabit the oral cavity, forming a biofilm whose main function is to protect against infection from other pathogenic microorganisms. Among the most representative phyla of the oral microbiome are *Streptococcus*, *Prevotella*, and *Veillonella* [41,42]. Oral bacteria contribute to nitric oxide metabolism through nitrate reduction, influencing vascular tone and blood pressure [43]. Furthermore, dysbiosis in the oral cavity can promote oxidative stress in periodontal tissues through pathogenic species such as *Porphyromonas gingivalis* which enhances macrophage ROS production [44].

### 3.5. The Microbiome in the Male and Female Reproductive Tracts

Both the male and female reproductive tracts contain complex microbial communities that play a fundamental role in homeostasis, including the oxidative balance and the reproductive processes. In women, the vaginal tract is mainly dominated by the *Lactobacillus genus*, which, in addition to providing an acidic pH and maintaining the vaginal mucosa, is characterized by producing hydrogen peroxide, promoting the existence of ROS [45]. This translates into an effective defense system against pathogens. When this group of bacteria is depleted, other genera such as obligate anaerobic bacteria *Prevotella*, *Gardnerella*, *Atopobium*, or *Sneathia* (associated with dysbiosis of the microbial community in the vaginal tract) increase [46], which can lead to vaginal bacteriosis, being considered a risk factor for infertility [45].

In men, the study of the semen microbiome has gained considerable interest thanks to new next-generation sequencing technologies which have shown that this niche is not completely sterile [47]. Approximately 30% of the microorganisms found in this environment come from the urethral microbiome [48]. The seminal microbiome contains a bacterial community characterized by genera such as *Lactobacillus*, *Gardnerella*, *Prevotella*, and *Pseudomonas* [49]. Many studies indicate that an imbalance in the microbial communities associated with semen is linked to subfertility. A greater abundance of Gram-negative bacteria has been described in the seminal microbiome of men with poorer semen-quality [48]. This group of bacteria possesses LPS, which can cause the release of pro-inflammatory cytokines [50]. This inflammatory environment can promote the fragmentation of sperm DNA, reducing its viability [51]. Figure 1 summarizes the main genera across the human body.

## 4. Microbiome-Mediated Modulation of Oxidative Stress

In addition to the functions outlined above, which are common to different human microbiomes, such as immunomodulation (e.g., expansion of regulatory T cells, production of antimicrobial peptides), maintenance of barrier integrity (expression of tight junctions, mucus production), and resistance to colonization by pathogens, the production of metabolites that can influence redox homeostasis and inflammatory pathways should be highlighted. Microbiome bacteria produce several secondary molecules which play a crucial role in relevant pathways (e.g., Nrf2 signaling), inducing antioxidant enzymes such as GPx, CAT, and SOD [60]. This is just one example of the regulatory role of the microbiome in redox homeostasis and its influence on the inflammatory processes.

Oxidative stress is increasingly recognized as being strongly influenced by the microbiome. Microbial metabolites and specific probiotic strains contribute to maintaining redox homeostasis, while interactions between diet and the microbiome further modulate this regulation. Several studies suggest the microbiome redox regulation through multiple pathways, such as regulating the immune system or influencing mitochondrial ROS production and antioxidant signaling pathways [2,61,62]. In this context, gut microbiome-derived metabolites have shown anti-inflammatory effects by promoting the differentiation of CD8^+^ tissue-resident memory T cells, mucosal-associated invariant T cells, and gut-resident regulatory T cells, with nitric oxide emerging as a key redox mediator in the regulation of T-cell function and gut-inflammatory processes [61]. On the other hand, the microbiome–gut–brain communication has been proposed to be influenced by SCFAs, affecting psychological functioning through G protein-coupled receptors, histone deacetylases, and humoral, hormonal, immune, and neural pathways [63]. Additionally, the microbiome plays a multifaceted role in modulating systemic inflammation (e.g., through cytokine signaling) and neuroinflammation (e.g., synthesis and metabolism of neurotransmitters such as serotonin, dopamine and GABA, or the maintenance of the blood–brain barrier’s integrity) [2]. On the other hand, the microbiome may influence mitochondrial function through molecular pathways involved in ROS production and inflammatory signaling, such as Nrf2/Keap1 and NF-κB [2,62].

### 4.1. Microbiome-Derived Metabolites with Antioxidant Properties

To date, SCFAs are the secondary metabolites produced by the microbiome that more frequently have been attributed to anti-inflammatory properties. These molecules, which include acetate, butyrate, and propionate, regulate fundamental processes such as reducing the accumulation of ROS, upregulating antioxidant enzymes (SOD, CAT, GPx), and reducing oxidation produced by the tumor necrosis factor-α signaling pathway [64,65,66].

In addition to SCFAs, there are other secondary metabolites from the microbiome that play an important role in regulating oxidative stress. Metabolites derived from tryptophan exhibit radical-scavenging properties, inhibit lipid peroxidation, and can modulate oxidative stress-related diseases in inflammatory models [67]. A great example is the indole-derived metabolites (indole 3-acetic acid, indole-3-propionic acid and indole-3-carboxaldehyde). These molecules can modulate the inflammatory response of colon tissue in inflammatory bowel disease (IBD) via the aryl hydrocarbon receptor (AhR) pathway [68].

The gut microbiome in the colon has been reported to be able to produce polyamines [69]. Polyamines (such as spermidine and cadaverine) can stabilize mitochondrial membranes and enhance autophagy, mitigating ROS accumulation [70]. Also, the microbial biotransformation of dietary polyphenols, which takes part in the colon, enhances their bioavailability, thereby increasing their antioxidant capacity [71].

### 4.2. Probiotic Strains with Antioxidant Effects: Lactobacilli, Bifidobacteria and Next-Generation Probiotics

Because bacteria have their own defense mechanisms against oxidation, they have the potential to regulate host redox balance. Lactobacilli and bifidobacteria have been used in several studies where their relationship with protection against oxidative stress has been demonstrated [72,73]. These bacterial families can contribute to antioxidant defense through distinct pathways. One of their antioxidant properties is the capability to chelate metal ions. These ions are capable of producing peroxyl and alkoxy radicals from hydrogen peroxide, then triggering lipid peroxidation. This lipid oxidation can produce cellular damage and has been linked to several diseases [74]. Thus, *Lactobacillus* spp. and *Bifidobacterium* spp. mitigate these processes. The *Lactobacillus* species *L. rhamnosus* GG and *L. paracasei* Fn032 have been reported to inhibit the formation of H_2_O_2_ from metal ions [75]. In addition, the antioxidant potential of certain proteins from *Bifidobacterium animalis* has been demonstrated. In particular, the proteins Pro-CK and Pro-Se were isolated from the strain *B. animalis* 01 and demonstrated antioxidant potential in various tests (reduction potential assay, erythrocyte hemolysis assay and 2,2-diphenyl-1-picrylhydrazyl radical-scavenging assay) [76]. Several lactobacilli have been reported to produce antioxidative enzymes which play a critical role in the host redox balance. In particular, SOD enzymes that use manganese as a cofactor and can break down superoxide anion into O_2_ and H_2_O_2_, have been found in several species of the genus *Lactobacillus*: *L. sakei*, *L. paraplantarum*, *L. buchneri*, *L. casei* and *L. brevis*.

Furthermore, next-generation probiotics such as *Faecalibacterium prausnitzii* and *Akkermansia muciniphila* show antioxidant and anti-inflammatory effects by producing metabolites that can regulate mitochondrial function and modulate the NF-κB signaling cascade [77,78,79].

### 4.3. Diet–Microbiome Interactions Influencing Redox Balance

Diet is the main determinant of the availability of nutrients that can be fermented by the gut microbiome, in addition to micronutrients, and therefore of the production of compounds that can affect the body’s redox potential [80]. The ability of SCFAs to regulate redox potential has already been mentioned, especially butyrate, as they modulate antioxidant pathways (Nrf2) and improve mitochondrial function by reducing ROS produced by the action of cytokines. Diets rich in fiber and polyphenols have a positive effect on the main bacteria that produce these SCFAs, such as *Anaerostipes* spp., *Eubacterium* spp., *Faecalibacterium prausnitzii*, and *Roseburia* spp. [81]. On the other hand, Western-style diets containing high saturated fats, simple sugars, and processed foods have been shown to reduce microbial diversity and promote the overgrowth of *Pseudomonadota*, leading to elevated ROS and systemic inflammation [82,83,84]. Several reports also highlight that antioxidant micronutrients such as selenium, zinc, and vitamins C and E modulate microbial composition and enhance host antioxidant responses [85,86,87,88]. Moreover, dietary supplementation with polyphenols can shift microbiome composition toward beneficial taxa and upregulate the host antioxidant defenses [89,90]. Thus, dietary modulation of the microbiome represents a promising tool for mitigating oxidative stress-related pathologies and improving systemic health.

## 5. Oxidative Stress as a Modulator of Microbiome Composition

Oxidative stress is increasingly recognized as a selective ecological pressure in the gut. It is a factor that can reshape the microbial community structure not merely by “damaging bacteria,” but by altering the intestinal redox landscape that determines which metabolic strategies are viable [91,92,93]. The healthy colon is characterized by low oxygen tension that supports obligate anaerobes and fermentation-based energy metabolism. When oxidative stress increases (via ROS, elevated epithelial oxygen leakage, or altered host oxygen-consumption) [94,95], the gut environment can shift toward relative hyperoxia and higher redox potential, which are conditions that systematically disadvantage strict anaerobes while favoring aerotolerant and facultative taxa capable of respiration or robust ROS detoxification [93]. This conceptual framework is supported by mechanistic work showing that host epithelial metabolism and oxygen availability act as key upstream determinants of microbial ecology, particularly during inflammatory states when oxygen and alternative electron acceptors become more available [96,97,98,99].

A key consequence of redox imbalance is a drift toward “dysbiosis-like” configurations characterized by a reduced abundance of butyrate-producing anaerobes and relative expansion of facultative organisms (often including *Pseudomonadota* and other aerotolerant groups) [100]. Butyrate producers are central to this story because butyrate fuels colonocyte respiration and helps maintain luminal anaerobiosis [101]. Loss of butyrate-producing communities can therefore create a permissive niche for oxygen-tolerant opportunists, reinforcing a self-perpetuating ecological loop in which diminished fermentation capacity and rising oxygen/redox potential further erode anaerobe dominance [100]. In parallel, inflammation generates ROS and nitrate, which can be exploited by *Enterobacteriaceae* via aerobic respiration and nitrate respiration, providing a mechanistic explanation for “blooms” of these taxa in inflamed ecosystems [97,98]. These shifts are not merely compositional; they alter microbial functional output (e.g., reduced fermentative metabolism, altered SCFAs production), with downstream effects on barrier integrity and immune tone [97,98].

Importantly, recent experimental evidence has begun to map which gut microbes and functions are most redox-sensitive in controlled systems [102]. In a cultivation-based study exposing human fecal microbiome communities and representative gut isolates to oxidative stress (oxygen and H_2_O_2_), oxidative challenge consistently reduced key butyrate-producing taxa (including genera such as *Agathobacter* and *Anaerostipes*) and depleted total butyrate output [6], while leaving many facultative anaerobes (e.g., members of *Escherichia-Shigella* and *Enterococcus*) comparatively unaffected. *Bacteroides* displayed notable resilience, highlighting that redox sensitivity is not uniform across major gut-lineages. The study also identified particularly sensitive taxa (e.g., *Fusicatenibacter saccharivorans* and *Lachnospira eligens*) and emphasized substantial inter-individual variability in community response [6].

Oxidative stress perturbs functional pathways that are tightly linked to host metabolic- and immune outcomes. Oxidative challenge can reduce carbohydrate fermentation and SCFAs production, shift carbon flow toward lactate and formate accumulation, and disrupt pathways such as succinate-to-propionate conversion in resilient taxa, illustrating that community-level “stability” may still mask meaningful metabolic rewiring [103]. Redox imbalance is not uniformly tolerated across taxa, creating a predictable ecological filter [104]. Many canonical butyrate-producing obligate anaerobes (often within *Clostridia*) are particularly vulnerable to oxygen/ROS stress [105], whereas facultative anaerobes can exploit the altered redox milieu. Conversely, certain “strict anaerobes” have evolved adaptations that partially buffer oxygen stress; for example, *Faecalibacterium prausnitzii* can use extracellular electron shuttling mechanisms linked to oxygen handling—highlighting that redox sensitivity is species- and pathway-specific rather than purely phylum-level [6,106]. At the systems level, perturbations that acutely raise gut redox potential (e.g., antibiotic exposure in experimental models) can rapidly restructure microbial communities, reinforcing redox as a proximal modulator rather than a slow, secondary correlate [107,108]. At the microbial gene/pathway level, inflammatory- and oxidative contexts are frequently associated with enrichment or upregulation of oxidative stress response programs (e.g., glutathione transport, cysteine biosynthesis, riboflavin-related metabolism), reflecting a broader reallocation of microbial resources toward survival under redox stress rather than toward metabolite production that typically supports mucosal homeostasis [103].

Taken together, these findings justify a more nuanced view of oxidative stress in microbiome science. ROS and oxygen are not just generic “harmful exposures,” but are parameters that reconfigure microbial competition by rewarding respiratory flexibility, detoxification capacity, and aerotolerance. Clinically and nutritionally, this implies that interventions aiming to restore eubiosis may need to consider redox ecology explicitly. Either by reducing pro-oxidant drivers (inflammation, barrier dysfunction, hyperoxia) or by supporting microbial functions that help re-establish anaerobiosis (e.g., fermentable substrates that promote butyrate-producing consortia). The emerging hyperoxia/redox dysbiosis literature further strengthens the idea that oxygen/ROS are tractable ecological levers with relevance across chronic-disease contexts, rather than niche phenomena restricted to acute inflammation [103,109].

## 6. Microbiome and Inflammation

The gut microbiome is deeply integrated into immune regulation, acting simultaneously as (I) a source of microbial-associated molecular patterns sensed by innate immune receptors, (II) a metabolic organ producing small molecules that tune host-immunity, and (III) an ecological community whose structure is itself shaped by inflammatory- and immune-mediated changes in the intestinal environment [110]. Under homeostatic conditions, host–microbe interactions promote barrier integrity and immune tolerance; however, when dysbiosis emerges, whether due to diet, antibiotics, infection, or host factors, immune signaling can shift toward chronic activation, barrier permeability may increase, and inflammatory cascades can become self-sustaining [99,111,112]. Reviews in this area emphasize that “microbiome–inflammation” is rarely a one-way pathway; instead, inflammation alters oxygen tension, nutrient availability, and antimicrobial pressures, which in turn select for different microbial communities, reinforcing or reshaping inflammatory trajectories [99,111,112].

One of the most compelling mechanistic motifs connecting dysbiosis to chronic inflammation is the oxygen and electron-acceptor model of inflamed-gut ecology. Inflammatory processes can increase epithelial oxygen leakage into the lumen and generate alternative electron acceptors (e.g., nitrate) [92]. These conditions selectively advantage facultative anaerobes, especially *Enterobacteriaceae* and other respiration-capable taxa, over obligate anaerobes that dominate in the healthy colon [98]. This shift is not simply a “marker” of inflammation, it can actively reprogram microbial metabolism toward respiratory growth and alter metabolite profiles in ways that influence mucosal immunity [113]. Experimental and conceptual syntheses have articulated how microbial respiration and redox metabolism contribute to *Enterobacteriaceae* expansion in inflamed settings, providing a mechanistic bridge between immune activation and community restructuring [92].

Within chronic inflammatory conditions, dysbiosis often features reduced microbial richness and depletion of butyrate-producing organisms alongside the overrepresentation of aerotolerant or pro-inflammatory-associated taxa [111,114]. These compositional shifts matter because they are coupled to metabolic shifts: reduced SCFA generation, altered bile-acid transformations, and changes in amino acid/tryptophan metabolism can impair the epithelial energy supply [115], thus weakening tight junction integrity, and alter immune cell programming [115,116]. The result is a plausible pathway whereby dysbiosis contributes to a chronic, low-grade inflammatory milieu through both barrier-dependent mechanisms (e.g., increased translocation of microbial products) and metabolite-dependent immune modulation [6].

Microbial metabolites are now widely viewed as immunomodulatory currencies that translate diet and microbial activity into host immune phenotypes [117,118]. Among the best-characterized are SCFAs, particularly butyrate and propionate, which influence epithelial barrier function and immune regulation through G protein-coupled receptor signaling and epigenetic mechanisms (including histone deacetylase inhibition), with downstream effects on regulatory T-cell differentiation and inflammatory tone [116,119]. Secondary bile acids represent another major axis, engaging host nuclear- and membrane receptors (e.g., the farnesoid X receptor and Takeda G protein-coupled receptor 5) that integrate microbial metabolism with host lipid/glucose handling and immune signaling [116]. Tryptophan-derived indoles (often acting via the aryl hydrocarbon receptor) can support barrier integrity and shape innate and adaptive immune responses [111]. Polyamines and other microbially derived lipid-like mediators further diversify this chemical dialog, collectively illustrating that “microbiome effects” on inflammation are frequently metabolite-mediated rather than taxa-mediated per se [111,116,117].

A crucial translational implication is that the microbiome’s role in inflammation cannot be reduced to single organisms or single pathways without losing biological realism [103]. The same taxon can be beneficial, neutral, or harmful depending on the substrate availability, community context, and host immune state. Likewise, the same inflammatory phenotype may arise from different ecological configurations that converge on similar metabolite deficits (e.g., low butyrate output) or similar barrier disruptions [120]. The oxidative stress dimension adds an additional layer: (I) inflammation-induced ROS and oxygen changes can directly suppress strict anaerobes and reduce butyrate-related functions, (II) simultaneously promoting aerotolerant taxa and reshaping immune-relevant metabolite pools. thereby coupling redox biology to inflammatory outcomes through predictable ecological selection pressures [99,112].

**Figure 2:**
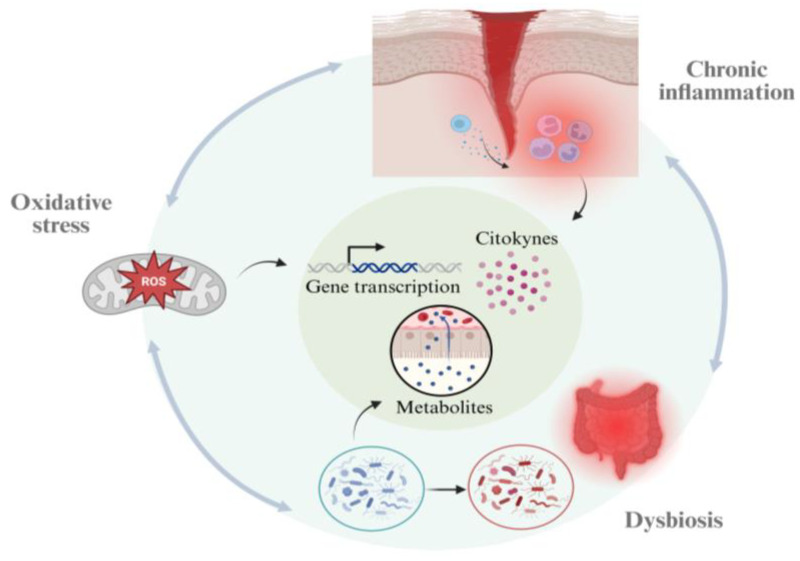
Interplay between microbiome, inflammation and oxidative stress.

In sum, microbiome–immune interactions should be conceptualized as a dynamic triad of community structure, metabolic output, and host inflammatory state, with feedback loops operating through redox ecology, barrier function, and immunometabolic signaling. This framing also clarifies why clinical translation increasingly focuses on restoring functions (e.g., SCFAs production, bile-acid balance, indole signaling) and stabilizing ecological conditions (anaerobiosis, substrate availability) rather than attempting to “add one probiotic strain” to correct a system-level inflammatory phenotype [6]. Overall, this complex interplay between microbiome, inflammation and oxidative stress is graphically schematized in Figure 2.

## 7. Clinical and Pathological Implications of the Triangular Interplay of Microbiome, Oxidative Stress, and Inflammation

### 7.1. Intestinal and Inflammatory Disorders

Dysregulation of the microbiome, as well as oxidative- and inflammatory conditions are a common finding in several intestinal disorders. For instance, the disturbance of the microbiome–oxidative stress–inflammation axis is particularly evident in IBD, where dysbiosis may promote diarrhea, colonic inflammation, oxidative stress, and pyroptosis [121]. Excessive ROS production reshapes microbial ecology, favoring taxa enriched in antioxidant enzymes such as glutathione reductase, perpetuating dysbiosis [122].

The main types of IBD are ulcerative colitis and Crohn’s disease. In ulcerative colitis, excessive ROS activate NF-κB and the NLR family pyrin domain containing 3 inflammasome, amplifying cytokine release and sustaining chronic inflammation [123]. Heightened oxidative stress and impaired antioxidant defenses act synergistically with inflammation to disrupt barrier integrity [124]. ROS-driven lipid peroxidation, epithelial injury, and leukocyte infiltration further reinforce mucosal inflammation [125]. In contrast, Crohn’s disease involves genetic- and epigenetic alterations in redox-regulating pathways (NOS2A, NOX1, DUOX2, NRF2, HIF1A) that exacerbate ROS production, compromise barrier function, and alter host–microbiome interactions [126]. Interestingly, fecal microbiome transplantation in patients with IBD has been shown to restore microbial balance and attenuate these processes [121].

Beyond IBD, other inflammatory disorders, such as systemic lupus erythematosus, exemplify how dysbiosis and oxidative stress converge to drive systemic disease. Nicotinamide adenine dinucleotide phosphate oxidase- and TLR7-mediated ROS production aggravates vascular dysfunction, while dysbiosis promotes Th17 polarization and autoimmunity in systemic lupus erythematosus [127]. Similar patterns occur across autoimmune diseases, where redox imbalance, barrier dysfunction, and microbial alterations sustain chronic inflammation [128].

In endometriosis, gut dysbiosis promotes oxidative stress and systemic inflammation, contributing to infertility and chronic pelvic pain. Microbial metabolites influence both the gut–vagina and gut–brain axes, driving neuroimmune activation and central sensitization, while hormonal dysregulation through the hypothalamic–pituitary–ovarian and hypothalamic–pituitary–adrenal axes amplifies redox imbalance [129]. Another example is psoriasis, where reduced SCFAs production due to dysbiosis impairs gut–brain communication, linking skin inflammation with psychiatric comorbidities such as depression and anxiety [130].

### 7.2. Metabolic Disorders

Metabolic disorders are related to the disturbance of several biochemical processes that lead to higher oxidative stress. In the context of obesity, an increase in O_2_^−^ production via oxidative phosphorylation, protein kinase C activation and glyceraldehyde auto-oxidation has been reported. This relationship is bidirectional, with oxidative stress promoting fat deposition and adipocyte growth and differentiation [131].

In other pathologies such as diabetes mellitus, hyperglycemia induces ROS overproduction, activates NF-κB, and drives cytokine release, generating a self-perpetuating oxidative–inflammatory loop that may be aggravated by dysbiosis [132]. Some common microbial alterations have been observed in diabetes mellitus and atherosclerosis, including enrichment of *Lactobacillus*, lipopolysaccharides (LPS), and trimethylamine N-oxide (TMAO), which impair endothelial function and induce vascular inflammation. In contrast, protective taxa such as *Bifidobacterium* mitigate these effects [133].

Liver metabolic disorders highlight the gut–liver axis. Dysbiosis and increased permeability allow LPS translocation, promoting hepatic lipid peroxidation and metabolic dysfunction. By contrast, SCFAs preserve barrier integrity and restore systemic balance [134].

Kidney diseases illustrate the convergence of the microbiome–oxidative stress–inflammation axis. In cisplatin-induced acute kidney injury and chronic kidney disease, tubular injury, systemic inflammation, and uremic toxin accumulation are exacerbated by dysbiosis [135,136]. Antibiotic-mediated depletion of the microbiome reduces cisplatin hepatotoxicity, confirming microbial involvement in redox imbalance and inflammatory activation [137]. Loss of SCFAs and increased uremic toxins promote oxidative stress, inflammation, and chronic kidney disease progression, while sodium butyrate restores redox balance and barrier integrity, attenuating kidney inflammation. In vitro, SCFAs reduce intracellular ROS and MDA while increasing SOD, underscoring their protective role against oxidative renal injury [138].

In heart failure, bowel-wall edema and hypoperfusion compromise the barrier integrity, promoting bacterial translocation and endotoxemia that sustain systemic oxidative- and inflammatory stress [139].

### 7.3. Neurodegenerative Disorders

The gut–brain axis plays a pivotal role in neurodegeneration. In Parkinson’s disease and Alzheimer’s disease, gut dysbiosis drives systemic inflammation, neuroinflammation, and oxidative stress, mediated by inflammasome activation, blood–brain barrier disruption, and altered SCFAs signaling [140].

Parkinson’s disease frequently begins with intestinal alterations preceding motor symptoms such as rigidity, tremors, and bradykinesia. Dysbiosis enhances intestinal permeability, oxidative stress, and α-synuclein aggregation, promoting immune activation and neuroinflammation [141,142]. Microbial metabolites further shape Parkinson’s disease progression: butyrate and indoles exert anti-inflammatory and neuroprotective effects, whereas excessive propionate and host-driven kynurenine pathway activation induce ROS accumulation, α-synuclein aggregation, and dopaminergic neurodegeneration [143].

In Alzheimer’s disease, amyloid-β aggregation, tau tangles, and synaptic dysfunction perpetuate oxidative stress and neuroinflammation, processes exacerbated by gut dysbiosis and elevated TMAO [112,144]. SCFAs such as butyrate mitigate ROS and inflammation through histone deacetylase inhibition and microglial modulation, while lauric acid improves mitochondrial function and reduces amyloid deposition. Together, these fatty acids provide epigenetic and metabolic support, offering a multifaceted strategy to slow Alzheimer’s disease progression [145].

### 7.4. Cancer

Cancer illustrates the convergence of microbiome, oxidative stress, and inflammation. Dysbiosis and in some cases its metabolites (e.g., TMAO, SCFAs, bile acids, LPS, and branched-chain amino acids) promote oxidative imbalance, immune dysregulation, and a pro-inflammatory tumor microenvironment [146]. For that reason, dysbiosis could contribute to carcinogenesis through multiple interconnected mechanisms: chronic inflammation, genotoxic metabolite production, and disruption of redox homeostasis. Importantly, specific microbial signatures have been reproducibly associated with distinct cancer types, advising that these are tumor-specific microbiome–host interactions rather than a uniform oncogenic microbiome [147].

#### 7.4.1. Colorectal Cancer

CRC represents the most extensively characterized cancer–microbiome association [148]. Enrichment of *Fusobacterium nucleatum* has been consistently reported in colorectal tumors and tumor progression, immune evasion, and poor prognosis. Mechanistically, *F. nucleatum* promotes carcinogenesis by activating β-catenin signaling, inducing pro-inflammatory responses, impairing antitumor immunity via interaction with immune inhibitory receptors [148]. In parallel, enterotoxigenic Bacteroides fragilis has been shown to induce DNA damage through ROS generation and chronic Th17-mediated inflammation, directly linking dysbiosis to redox imbalance and genomic instability. Reduced abundance of butyrate-producing taxa (e.g., *Faecalibacterium prausnitzii*) further compromises epithelial integrity and anti-inflammatory signaling [149]. Dysbiosis further decreases SCFAs production and increases carcinogenic metabolites such as secondary bile acids and TMAO, fueling DNA damage, barrier disruption, and inflammation. Lipid peroxidation products including MDA and 4-hydroxy-nonenal serve as biomarkers of oxidative imbalance in CRC progression and recurrence [150]. Indole-3-acetic acid, a tryptophan-derived metabolite, exemplifies this duality. Indole-3-acetic acid can generate ROS and exert anti-inflammatory effects, but via AhR activation, it fosters immune suppression and tumor progression [151].

#### 7.4.2. Gastric Cancer

In gastric cancer, *Helicobacter pylori* remain the most established microbial carcinogen. Beyond its inflammatory effects, *H. pylori* infection induces oxidative stress, DNA damage, and epigenetic alterations in gastric epithelial cells [152]. Recent studies indicate that gastric dysbiosis extends beyond *H. pylori*, with shifts toward nitrate-reducing and pro-inflammatory bacterial communities [153]. These changes may contribute to carcinogenesis through nitrosative stress and altered redox balance [153].

#### 7.4.3. Hepatocellular Carcinoma

The gut–liver axis plays a central role in hepatocellular carcinoma development. Dysbiosis characterized by increased abundance of endotoxin-producing Gram-negative bacteria and reduced beneficial commensals promotes intestinal permeability and chronic hepatic inflammation via LPS-mediated TRL signaling [154]. These processes exacerbate oxidative stress and fibrotic remodeling, creating a permissive environment for malignant transformation [155]. Altered bile-acid metabolism driven by the gut microbiome further modulates hepatic immune responses and redox signaling [156].

#### 7.4.4. Breast Cancer

Emerging evidence links gut-microbiome composition to estrogen metabolism and breast-cancer risk. Dysbiosis-associated increases in bacterial β-glucuronidase activity can enhance enterohepatic recirculation of estrogens, thereby increasing systemic estrogen exposure [157]. Additionally, altered microbial diversity has been associated with systemic inflammation and oxidative stress, potentially influencing the tumor microenvironment and disease progression [158].

#### 7.4.5. Pancreatic and Other Extraintestinal Cancers

Distinct intratumoral microbiome profiles have been identified in pancreatic ductal adenocarcinoma, with certain bacterial taxa associated with long-term survival and enhanced antitumor immune responses [159]. Experimental models suggest that tumor-associated bacteria can modulate oxidative metabolism and immune-cell infiltration, influencing tumor growth and responsiveness to therapy [160].

## 8. Therapeutic and Diagnostic Potential

A therapeutically useful way to think about redox–microbiome crosstalk is as a bidirectional control system: oxidative stress reshapes microbial ecology (often via oxygen/redox shifts), while microbial metabolism can either buffer or amplify host redox tone through effects on barrier integrity, immune signaling, and the generation (or depletion) of antioxidant-related metabolites [91]. This framing makes “antioxidant therapy” more than a host-directed strategy; it becomes a means of altering the ecological constraints that determine which microbes thrive and which functions dominate. Mechanistic studies show that epithelial-derived ROS can be degraded into molecular oxygen within the gut lumen, providing an energetic advantage to respiration-capable bacteria such as *E. coli* [161,162]. Complementary work demonstrates that inflammatory host chemistry (e.g., nitrate generation) can fuel blooms of facultative organisms, reinforcing that “oxidative/inflammatory” environments are not only damaging but also selectively permissive for certain microbial strategies [163].

### 8.1. Antioxidant Therapies That Modulate the Microbiome

Diet-derived antioxidants, polyphenols, carotenoids, antioxidant vitamins, and bioactive peptides, have been repeatedly linked to shifts in microbiome composition and function in experimental and clinical contexts, often with enrichment of taxa and pathways associated with saccharolytic fermentation and SCFAs generation. While the field is heterogeneous (diet matrices, doses, baseline diets, and sequencing platforms vary widely) [164,165], the overarching signal is that many antioxidant-rich interventions behave as “microbiome modulators,” in part because polyphenols and related compounds reach the colon where microbial biotransformation generates smaller, often more bioactive metabolites that can feed back on microbial ecology and host redox defenses [164,165].

In line with this, a recent systematic review and meta-analysis of randomized trials in adults who are overweight/obese found that polyphenol-rich interventions can modify the gut microbiome and are associated with changes in oxidative stress/antioxidant defense markers [166], supporting the idea that antioxidant supplementation can operate through combined host–microbe pathways rather than purely systemic antioxidant effects [166].

Classical pharmacologic antioxidants have also been shown to remodel the microbiome in preclinical models. Melatonin, for instance, has been reported to improve oxidative stress resistance and modulate intestinal microbial communities in experimental colitis, consistent with its combined antioxidant and immunomodulatory actions [167]. *N*-acetylcysteine has likewise been shown to improve gut redox status and influence the microbiome in stress models, offering a mechanistic bridge between redox buffering and microbial community shifts [168,169]. Although much of this evidence is not yet anchored in large human trials, it provides a biologically coherent rationale. Interventions that reduce mucosal oxidative pressure may help stabilize the ecosystem and support the recovery of strict anaerobes and their fermentation outputs.

A particularly translationally attractive direction is targeting host redox signaling hubs that sit at the intersection of oxidative defense, barrier integrity, and microbial selection pressures, especially the Nrf2 axis. Notably, gut-resident lactobacilli have been shown to activate hepatic Nrf2 and confer protection in vivo, illustrating that microbial or microbe-derived factors can engage host-antioxidant programs and potentially feedback to shape the intestinal environment [170]. Future interventions can be designed to deliberately co-opt host redox signaling and microbial ecology as an integrated system.

### 8.2. Lifestyle Modifications as Foundational Therapy for Dysbiosis

Lifestyle factors are upstream determinants of how gut microbial ecology and function could change, with this done, in part, by shaping substrate availability, bile acid and SCFA production, barrier integrity, and inflammatory–oxidative tone [171]. Therefore, lifestyle optimization should be framed as a foundational layer for dysbiosis-oriented strategies, particularly in conditions characterized by oxidative stress and chronic inflammation. This is clinically relevant because the magnitude and durability of the response to microbiome-targeted interventions may be constrained by an adverse host ecological context. Also, this can perpetuate redox imbalance and favor facultative, stress-tolerant microbial strategies.

#### 8.2.1. Weight Loss and Adiposity Reduction

Excess adiposity contributes to chronic low-grade inflammation and oxidative stress [172]. This has been linked to unfavorable shifts in gut microbial ecology, reduced diversity, and lower production of beneficial metabolites like SCFAs [173]. Weight loss through dietary energy restriction or bariatric surgery has been shown to modify the gut microbiome [174] by increasing diversity and beneficial taxa, likely through reducing systemic inflammation and altering metabolic signaling [174]. A 2022 systematic review and meta-analysis reported these previous assumptions and add reductions in intestinal permeability. The systematic review supports the concept that reducing adiposity-related inflammatory/oxidative burden can shift the intestinal ecosystem toward a more resilient state [175]. In severe obesity, bariatric surgery is also followed by reproducible microbial changes. Recent meta-analytic evidence synthesizing pre- and post-surgery data supports significant compositional shifts after metabolic/bariatric procedures. Consistent with sustained changes in host physiology and intestinal ecology accompanying a large weight loss [176].

#### 8.2.2. Dietary Pattern and Dietary Quality

Diet has the strongest evidence among lifestyle factors shaping microbial ecology [177,178]. Diets that are rich in plant-derived fibers and polyphenols favor saccharolytic fermentation and SCFA production, and have reported epithelial integrity and immune regulation [179]. Conversely, Western-diet patterns are associated with reduced diversity and an increase in pro-inflammatory taxa [180]. Controlled feeding studies show that diet can rapidly and reproducibly alter the human gut microbiome, reinforcing the idea that dietary pattern is an ecological control for microbial metabolism [181].

#### 8.2.3. Physical Activity

Exercise appears to improve gut-microbiome diversity in adults. A 2024 systematic review and meta-analysis reported increases in Shannon diversity, supporting physical activity as a practical lever to improve dysbiosis-related community features [182]. More recent integrative syntheses also emphasize bidirectional links between exercise, microbiome-derived metabolites (including SCFAs), and host-immunometabolic signaling, which is mechanistically consistent with improved barrier function and reduced pro-oxidant inflammatory tone [183,184].

#### 8.2.4. Sleep and Circadian Alignment

Sleep disruption and circadian misalignment are increasingly recognized as contributors to microbiome perturbation and metabolic dysregulation [185,186]. Recent systematic reviews focusing on shift circadian disruption describe consistent associations with altered microbiome profiles, providing a clinical rationale to address circadian factors as part of dysbiosis management—particularly in chronic inflammatory contexts [185,186].

#### 8.2.5. Environmental Exposures and Risk Behaviors

Environmental exposures that increase oxidative stress may also destabilize the gut ecosystem. Recent reviews synthesize evidence that air pollution can perturb the gut microbiome and host–microbe signaling, positioning exposure reduction as a plausible enabling condition for microbiome-directed therapies [187,188]. In parallel, tobacco exposure is consistently linked to microbiome alterations. Large-scale human evidence continues to accumulate that smoking-related microbial patterns are associated with metabolic outcomes, in addition to smoking cessation as a relevant component of ecological correction [189,190]. For alcohol, recent meta-analytic work has evaluated ethanol-associated microbiome changes, supporting the concept that chronic ethanol exposure promotes dysbiosis and barrier dysfunction, factors that may sustain oxidative/inflammatory loops [191].

Lifestyle optimization should be presented as the first therapeutic layer that reduces pro-oxidant drivers and restores conditions supportive of anaerobic fermentation and barrier integrity. This framing helps explain heterogeneity across probiotic/biologic trials: colonization, functional output, and downstream redox benefits may be limited when the underlying ecological context remains unfavorable.

### 8.3. Probiotics, Prebiotics, and Synbiotics in Redox Regulation

Probiotics and synbiotics are increasingly evaluated using oxidative-stress biomarkers as outcomes (e.g., SOD, total antioxidant capacity), reflecting recognition that redox status is a clinically relevant intermediate phenotype in metabolic and inflammatory disorders. Meta-analytic evidence across metabolic disease contexts suggests that probiotic supplementation can improve oxidative stress and inflammatory biomarkers, although effect sizes vary by population, strain selection, dose, and duration, underscoring that “probiotic” is not a single intervention class but a family of strain-specific and context-dependent tools [192,193]. Importantly, synbiotics may offer an ecological advantage by pairing strains with fermentable substrates that enhance colonization efficiency and functional output; moreover, potentially strengthening effects on redox buffering through increased SCFAs production and improved barrier function.

An illustrative clinical example is a randomized, placebo-controlled trial in adults with metabolic syndrome showing that 12 weeks of daily synbiotic yogurt consumption improved multiple oxidative stress status measures, providing direct human-evidence that combined microbial–substrate interventions can move redox endpoints in a high-risk phenotype [194]. While many such trials do not comprehensively profile microbiome function, their biomarker improvements justify deeper mechanistic studies that connect specific microbial shifts and metabolite outputs to redox benefits.

Prebiotics and dietary fibers represent another redox-relevant lever because they can restore fermentative metabolism that acidifies the lumen and supports anaerobiosis, conditions generally unfavorable to oxidative metabolism-adapted opportunists. A compelling demonstration comes from research showing that fiber supplementation protects against antibiotic-induced dysbiosis by modulating gut redox potential, supported by metatranscriptomics and direct chemical measurements of redox and pH [195]. This is an important proof-of-concept for “redox-directed microbiome therapy”. Rather than only adding microbes, one can reshape the physicochemical niche (redox potential) to preserve or restore commensal function.

### 8.4. Microbial Markers of Oxidative Stress as Diagnostic Tools

On the diagnostic side, the redox concept opens two complementary biomarker avenues: (I) direct measurement of the gut physicochemical state (e.g., fecal redox potential and pH), and (II) microbiome-derived signatures (taxa, genes, and metabolites) that reproducibly track redox imbalance. The appeal of fecal redox/pH is that they can serve as functional proxies for ecosystem metabolism at the bedside; a longitudinal pilot study in preterm infants, for example, supports the feasibility of repeated pH and redox measurements and links these measures to SCFAs content and microbiome composition, positioning them as candidate “homeostasis monitors” during early colonization [196]. Similar ideas have been discussed for adult gut-health, and clinical perspectives increasingly argue for redox potential as a central hub linking diet, epithelium, and microbial ecology [197].

However, diagnostic optimism must be balanced with careful validation. In IBD, for instance, direct measurement of oxidation–reduction potential in fecal water did not demonstrate diagnostic value in one study, highlighting that sampling methods, matrix effects, and disease context can limit performance of redox measures as standalone diagnostics [198]. This argues for composite approaches that combine redox readouts with microbial and metabolite signatures.

Microbiome-based diagnostics are already advancing rapidly in inflammatory disease. A recent study developed a multibacteria biomarker panel (implemented via multiplex droplet digital PCR) with strong discriminative performance for IBD [199]. Translating this logic to oxidative stress would mean building panels around (I) redox-sensitive ecological shifts (e.g., loss of strict anaerobes and enrichment of respiration-capable taxa) and (II) functional modules tied to oxidative defense and redox metabolism. Emerging work that links measured oxidation–reduction potential in mucosal tissue to characteristic microbiome changes further supports the feasibility of “redox–microbiome coupled” biomarkers, though larger cohorts are needed [196,200]. Finally, methodological advances in redoxomics, including low-input workflows that enable broader profiling of redox-active metabolites, create an enabling layer for identifying microbial or host-derived redox markers that could be paired with metagenomics/metabolomics for more specific diagnostics [201,202].

### 8.5. Role of Microbiome in the Efficacy of Immunotherapies

Beyond this relationship between the microbiome and immune response, the potential role of the microbiome in the improvement of immunotherapies has gained attention, with preclinical models showing promising results [203]. This evidence is currently growing in humans, with multiple studies supporting the idea that the microbiome may profoundly influence the efficacy of immunotherapy, as well as other therapies where the immune system has a relevant role (e.g., chemotherapies with immunostimulatory functions) [204]. Immunotherapy provides an innovative approach that generally combines two strategies, an induction of a direct immune response and a reactivation of antitumor immunity, thus harnessing the immune system to combat tumor cells. The mechanisms by which the microbiome can have an impact in immunotherapy efficacy are diverse, including influencing the tumor microenvironment, activating pattern-recognition receptors, molecular mimicry (capacity of producing antigens recognizable by host immune cells), or microbial metabolites with modulatory functions [203,204].

Among others, the microbiome has been associated with outcomes of immune checkpoint inhibitors. For instance, in unresectable hepatocellular carcinoma, the coexistence of *Prevotella* 9 depletion and *Lachnoclostridium* enrichment predicted better overall survival [205]. The influence on anti-programmed cell death 1 protein immunotherapy has also been reported. For example, in the treatment of melanoma, the gut microbiome of responders showed significantly higher alpha diversity and relative abundance of the *Ruminococcaceae* family [206]; or in the treatment of prostate cancer, a reduction in *Akkermansia muciniphila* in responders has been observed [207]. These are just a few examples, there are several existing clinical trials investigating these interactions and evaluating different strategies (prebiotics, probiotics, fecal microbiome transplantation) to improve the efficacy of immunotherapy through changes in the microbiome composition [204].

## 9. Challenges and Future Directions

### 9.1. Causality and the Human Translation Gap

A central limitation of the redox–microbiome literature is that mechanistic clarity often comes from animal- or ex vivo systems, whereas clinical relevance is demanded in humans where diet, medications, host genetics, comorbidity, and environment introduce strong confounding variables. Even when associations are robust, directionality is hard to infer: oxidative stress can drive dysbiosis, but dysbiosis can also increase oxidative stress through barrier dysfunction, immune activation, and altered metabolite profiles. The field therefore needs study designs that can adjudicate causality—e.g., longitudinal sampling with time-lagged modeling, controlled feeding trials, perturbation studies (antibiotics, fiber rescue), and interventions that explicitly target redox state and quantify microbial/ecosystem response. The broader clinical-translation literature on microbiome medicine emphasizes that trial design, standardization, and reporting quality remain major bottlenecks preventing reliable implementation, even when the biological rationale is strong [208,209].

### 9.2. Multi-Omics Integration, Including “Redoxomics”

Progress will likely depend on integrating who is there (metagenomics) with what they are doing (metatranscriptomics/proteomics) and what chemistry they produce (metabolomics), and, increasingly, with redox-focused molecular layers. Foundational frameworks have long argued that combining metagenomics, metatranscriptomics, and metabolomics yields a more complete functional picture than any single layer [210,211]. Recent methodological reviews highlight both the promise and the pitfalls of multi-omics integration (batch effects, compositionality, annotation limits, and cross-platform harmonization), making it clear that analytical rigor is not optional if the goal is clinical-grade biomarkers [212,213].

Redoxomics offers an additional axis that is particularly relevant to oxidative-stress biology because it can capture redox-active metabolites and oxidative modifications that are not well represented in standard untargeted metabolomics. Low-input redoxomics methods and redox systems biology frameworks are beginning to provide scalable ways to map redox landscapes in complex biological systems, which can be paired with microbiome profiles to identify redox-sensitive microbial functions and redox-linked community states [201,202,214]. The immediate opportunity is not just discovery, but validation: establishing which redoxomic features are stable, which are diet/medication-sensitive, and which add predictive value beyond conventional inflammatory or oxidative markers.

### 9.3. Systems Biology and AI to Predict Microbiome–ROS Interactions

Because microbiome–ROS interactions are inherently networked (feedback between immunity, epithelial metabolism, microbial respiration/fermentation, and metabolite signaling), systems biology approaches are well-positioned to move the field from descriptive associations to mechanistic, testable models. Genome-scale metabolic modeling and multi-scale computational frameworks have been proposed as routes to integrate host metabolism with microbial metabolism and to simulate how environmental constraints (including redox availability) reshape pathway fluxes and community structure [215,216].

Artificial intelligence and machine learning can add value, but only if deployed with discipline. Reviews and best-practice frameworks emphasize that microbiome machine-learning is vulnerable to overfitting, batch effects, leakage across folds, and poor external validity; interpretability and rigorous validation (preferably external) are essential for models intended for biomarker discovery or clinical prediction [217,218,219]. The most productive near-term use case may be interpretable models that integrate microbiome plus metabolome plus redox readouts to identify minimal, transportable signatures (e.g., small marker panels plus a few redoxomic metabolites) that generalize across cohorts. Explainable artificial-intelligence approaches applied to multi-omics disease discrimination illustrate the direction of travel, though larger and more diverse datasets are still required to avoid “context-specific” models [199,220,221].

### 9.4. Need for Longitudinal and Interventional Studies

Finally, the redox–microbiome field needs more time in its designs. Longitudinal sampling is indispensable for distinguishing transient shifts (e.g., after antibiotics) from durable ecological remodeling and for identifying whether redox changes precede compositional changes or vice versa. Intervention studies that directly manipulate redox constraints are particularly valuable because they can provide causal leverage. Fiber supplementation that dampens antibiotic-induced increases in gut redox potential represents a strong template: it couples a defined perturbation, repeated physicochemical measurements, and multi-omic readouts to identify mechanisms and outcomes [195,196]. Extending this logic to clinical populations (metabolic disease, IBD, liver disease) with standardized redox measurement protocols and harmonized multi-omics pipelines is the clearest path toward robust, clinically usable microbiome–ROS diagnostics and therapies.

## 10. Conclusions and Translational Outlook

The interaction between the human microbiome, oxidative stress, and inflammation is increasingly recognized as a dynamic, bidirectional system rather than a one-way pathway. Oxidative- and inflammatory pressures alter the physicochemical environment of mucosal surfaces, particularly oxygen tension and redox potential, thereby selecting for microbial traits such as aerotolerance, respiratory flexibility, and stress-response capacity. In parallel, the microbiome shapes host redox balance through its metabolic output and strain-specific functions, influencing epithelial barrier integrity, immune signaling, and systemic inflammatory tone. This reciprocity helps explain why microbial changes observed in disease often reflect not only shifts in community membership but also functional remodeling under redox stress.

This framework has important implications for both physiology and pathology. In health, microbial metabolites and host–microbe crosstalk support redox homeostasis and immune tolerance by sustaining anaerobic fermentation, reinforcing barrier function, and generating immunomodulatory molecules. In disease-prone contexts, redox imbalance can drive ecological drift toward configurations with reduced fermentative capacity and altered metabolite profiles, weakening mucosal defenses and sustaining low-grade inflammation. Critically, similar clinical phenotypes may arise from distinct microbial configurations that converge on shared functional deficits (e.g., reduced SCFAs production or disrupted bile acid and tryptophan-derived signaling), supporting a shift from taxonomic descriptions to function-centered interpretation and intervention.

Translationally, the microbiome–oxidative stress axis represents a tractable target if approached as an ecological system with measurable constraints and outputs. Progress will depend on standardized phenotyping of redox status, longitudinal designs that clarify directionality, and interventions that explicitly modify redox conditions while tracking microbial- and host responses. Integrative multi-omics, including redox-focused molecular layers, will be essential to identify robust biomarkers and minimal signatures that generalize across populations. Ultimately, therapies that restore anaerobic metabolism, strengthen barrier integrity, and rebalance immunometabolic signaling, through diet, targeted microbial therapies, and combined lifestyle strategies, offer a promising route to convert mechanistic understanding into clinically usable diagnostics and treatments.

## Figures and Tables

**Table 1:** Main species of ROS/RNS [7].

Free radical species	
Superoxide anion radical	O_2_^•−^
Hydroxyl radical	^•^OH
Alkoxyl	^●^OR
Nitric oxide	NO^•^
Peroxyl radicals	^●^OOR
Non-free radical species	
Hydrogen peroxide	H_2_O_2_
Nitrogen dioxide	NO_2_
Peroxynitrite	ONOO^−^

**Table 2:** Components and functions of the antioxidant defense system [11,12,13].

Enzymatic components	
SOD	Catalyzes the dismutation of Superoxide anion radical: O_2_^•−^ ➔ O_2_ + H_2_O_2_
GPx	Reduction in lipid peroxides or H_2_O_2_ to alcohols and H_2_O: R-OOH + 2GSH ➔ R-OH + H_2_O + GSSG
CAT	Decomposes Hydrogen peroxide: H_2_O_2_ ➔ H_2_O + O_2_
TrxR	Reduction in a variety of radicals such as lipid hydroperoxides, or protein thiols
Non-enzymatic components	
Vitamin C	Acts as radical scavenger reacting with a different ROS/RNS species and redox-active transition metals Regeneration of vitamin E from radical tocopheryl form
Vitamin E	Radical scavenger through the donation of the H atom from its hydroxyl group with the subsequent conversion in radical tocopheryl
Flavonoids	Polyphenolic compounds with the ability to scavenge ROS/RNS forming flavonoid phenoxyl radicals or to chelate redox metal ions
Carotenoids	Neutralize ROS/RNS through their interaction with the system of conjugated double bonds

Abbreviations: CAT, catalase; GPx, glutathione peroxidase; GSH, reduced glutathione; GSSG, oxidized glutathione; H, hydrogen; H_2_O, water; H_2_O_2_, hydrogen peroxide; O_2_, oxygen; O_2_^•−^, superoxide anion radical; R-OH, alcohols; R-OOH, peroxides, SOD, superoxide dismutase; TrxR, thioredoxin reductase.

## Data Availability

No new data were created or analyzed in this study.
